# Mixture models for analyzing product reliability data: a case study

**DOI:** 10.1186/s40064-015-1420-x

**Published:** 2015-10-22

**Authors:** S. Ruhi, S. Sarker, M. R. Karim

**Affiliations:** Department of Mathematics, Pabna University of Science & Technology, Pabna, Bangladesh; Department of Statistics, University of Rajshahi, Rajshahi, 6205 Bangladesh

**Keywords:** Case study, Data analysis, EM algorithm, Mixture model, Reliability

## Abstract

In the case of manufactured products, there are situations where some components of a product are produced over a period of time by collecting items from different vendors, using different raw materials, machines, and manpower. The physical characteristics and the reliabilities of such components may be different, but sometimes it is difficult to distinguish them clearly. In such situations, mixtures of distributions are often used in the analysis of reliability data for these components. Here a twofold Weibull–Weibull mixture model is applied to analyze product reliability data that consist of both failure and censored lifetimes. The Expectation–Maximization (EM) algorithm is used to find the maximum likelihood estimates of the model parameters. As a case study, it analyses an Aircraft component (Windshield) failure data and various characteristics of the mixture model, such as the reliability function, B10 life, mean time to failure, etc., are estimated to assess the reliability of the component. Simulation studies are performed to investigate the properties and uses of the proposed method.

## Background

Reliability of a product is defined by the probability that the product will perform well it is intended function for a specified time period or usage limit under normal operating condition (Meeker and Escobar 1998; Blischke and Murthy 2000). Because of rapid advances in manufacturing technology, customers expect to purchase products that will be highly sophisticated, reliable and safe. In recent years many manufacturers are collecting and analyzing field failure data to enhance the reliability of their products and to improve goodwill and customer satisfaction (Blischke et al. 2011).

The paper analyses an Aircraft component (Windshield of a particular model) failure data that contain both failure and censored times. The same data are analyzed by Murthy et al. (2004) and Ruhi (2015). Murthy et al. (2004) have fitted a twofold Weibull mixture model for the data and estimated the parameters of the model by applying a graphical method based on Weibull probability paper (WPP) plot. The graphical method has been used widely to estimate the parameters of the Weibull mixture model. The bulk of the existing literature deals with the well-separated subpopulations cases and uses various approximations for plotting and the characterizations of the asymptotes. As mentioned in Murthy et al. (2004), there are two serious drawbacks in the graphical method. These are as follows:

- The graphical method yields very crude estimates unless applied repeated iteration and evaluated by vision. As such, they can be used as starting points for more sophisticated statistical methods.
- They do not provide any statistical confidence limits for the estimated parameters.

To overcome these drawbacks, here we apply the Expectation–Maximization (EM) algorithm to find the maximum likelihood estimates of the twofold Weibull mixture model and investigate the performance of the proposed method over the method of Murthy et al. (2004). The performance of the method will be evaluated by numerical simulation studies.

The outline of the paper is as follows. “Mixture models” describes the assumed mixture model. “Maximum-likelihood estimation of model parameters” explains the parameter estimation method. “Case study: analysis of aircraft Windshield failure” data presents a case study based on aircraft Windshield failure data. “Simulation study” presents a simulation study to investigate the performance of the method and “Conclusion” concludes the paper. Finally Appendix provides R codes that used in the paper for estimating the parameters of the model.

## Mixture models

Various types of statistical models have been applied extensively in the analysis of failure data for manufactured products. However, there are situations where some components of a product are produced over a period of time by collecting items from different vendors, using different raw materials, machines, and manpower. In such situations, mixtures of distributions are often used in the analysis of reliability data as the physical characteristics and the reliabilities of such components may be different and difficult to distinguish easily and clearly.

The cumulative distribution function (cdf), probability density function (pdf) and hazard function (hf) of a general *K*-fold mixture model involving *K* subpopulations have presented in Murthy et al. (2004) and Blischke et al. (2011). The cumulative distribution function (cdf) of a general *K*-fold mixture model can be written as

1 $$G(t) = \sum\limits_{j = 1}^{K} {p_{j} F_{j} (t)}$$

where *F*_*j*_(*t*) is the cdf of the *j*-th sub-population, *p*_*j*_ is the mixing probability of the *j*-th sub-population, *p*_*j*_ > 0 and $\sum\nolimits_{j = 1}^{K} {p_{j} = 1}$. The probability density function (pdf) is given by

2 $$g\left( t \right) = \sum\limits_{j = 1}^{K} {p_{j} f_{j} \left( t \right)}$$

where *f*_*j*_(*t*) is the pdf associated with *F*_*j*_(*t*). The hazard function *h*(*t*) is

3 $$h\left( t \right) = \sum\limits_{j = 1}^{K} {w_{j} \left( t \right)\,h} {}_{j}\left( t \right)$$

where *h*_*j*_(*t*) is associated with subpopulation *j*, and

4 $$w_{j} \left( t \right) = \frac{{p_{j} R_{j} \left( t \right)}}{{\sum\limits_{j = 1}^{K} {p_{j} R_{j} \left( t \right)} }},\quad j = 1,2, \ldots ,K,$$

where $$\sum\nolimits_{j = 1}^{K} {w_{j} (t) = 1}$$ with

5 $$R_{j} \left( t \right) = 1 - F_{j} \left( t \right),\quad j = 1,2, \ldots ,K .$$

From (3), we see that the failure rate for the model is a weighted mean of the failure rate for the subpopulations with the weights varying with *t*, *t* ≥ 0.

### Special case: twofold Weibull mixture model

The cdf of the twofold mixture model (putting *K* = 2 in (1)) for the random variable *T* is given by

6 $$G\left( t \right) = pF_{1} \left( t \right) + \left( {1 - p} \right)F_{2} \left( t \right),\;\;t \ge 0$$

If *F*_1_(*t*) follows Weibull (*α*_1_, *β*_1_) and *F*_2_(*t*) follows Weibull (*α*_2_, *β*_2_) distributions, the cdf for twofold Weibull–Weibull mixture model from Eq. (6) becomes

7 $$G\left( t \right) = \left[ {1 - \exp \left[ { - \left( {\frac{t}{{\alpha_{2} }}} \right)^{{\beta_{2} }} } \right]} \right] +\,p\left[ {\exp \left\{ { - \left( {\frac{t}{{\alpha_{2} }}} \right)^{{\beta_{2} }} } \right\} - \exp \left\{ { - \left( {\frac{t}{{\alpha_{1} }}} \right)^{{\beta_{1} }} } \right\}} \right],\quad \{ \beta_{1} ,\alpha_{1} ,\beta_{2} ,\alpha_{2} \} > 0,\;\;t \ge 0$$

The corresponding probability density function (pdf) is

8 $$g\left( t \right) = p\left[ {\frac{{\beta_{1} }}{{\alpha_{1} }}\left( {\frac{t}{{\alpha_{1} }}} \right)^{{\beta_{1} - 1}} \exp \left( { - \frac{t}{{\alpha_{1} }}} \right)^{{\beta_{1} }} } \right] + \left( {1 - p} \right)\left[ {\frac{{\beta_{2} }}{{\alpha_{2} }}\left( {\frac{t}{{\alpha_{2} }}} \right)^{{\beta_{2} - 1}} \exp \left( { - \frac{t}{{\alpha_{2} }}} \right)^{{\beta_{2} }} } \right],\quad t \ge 0$$

The reliability function is

9 $$R\left( t \right) = p\left[ {\exp \left[ { - \left( {\frac{t}{{\alpha_{1} }}} \right)^{{\beta_{1} }} } \right]} \right] + \left( {1 - p} \right)\left[ {\exp \left[ { - \left( {\frac{t}{{\alpha_{2} }}} \right)^{\beta 2} } \right]} \right],\quad t \ge 0$$

And the hazard function is

10 $$h\left( t \right) = \frac{{pf_{1} (t) + (1 - p)f_{2} (t)}}{{p\exp \left[ { - \left( {\frac{t}{{\alpha_{1} }}} \right)^{{\beta_{1} }} } \right] + (1 - p)\exp \left[ { - \left( {\frac{t}{{\alpha_{2} }}} \right)^{{\beta_{2} }} } \right]}},\quad t \ge 0$$

Other two-fold mixture models can be derived by using different cdfs from different lifetime distributions, similarly. In the remainder of the paper we apply this twofold Weibull mixture model to analyze the data set.

## Maximum-likelihood estimation of model parameters

If the data contain the failure/censored random variable *t*_*i*_ and failure/censored indicator *δ*_*i*_, (if the *i*th observation is failure then *δ*_*i*_ = 1 and if it is censored then *δ*_*i*_ = 1) for $i = 1, 2, \cdots n$, the likelihood function under random censoring scheme for the data is given by

11 $$L(\theta ) = \prod\limits_{i = 1}^{n} {\left[ {f(t_{i} )} \right]^{{\delta_{i} }} \left[ {1 - F(t_{i} )} \right]^{{1 - \delta_{i} }} }$$

where, θ is the parameter vector for the assumed model. Taking log on both sides of Eq. (11), we get,

12 $$\ln L = \sum\limits_{i = 1}^{n} {\left[ {\delta_{i} \ln f\left( {t_{i} } \right) + \left( {1 - \delta_{i} } \right)\ln \left\{ {1 - F\left( {i_{i} } \right)} \right\}} \right]}$$

In the case of twofold Weibull mixture model with *θ* = {*β*_1_, *α*_1_, *β*_2_, *α*_2_, *p*}, the log-likelihood function (12) becomes

13 $$\begin{aligned} \ln L & = \sum\limits_{i = 1}^{n} {\delta_{i} \ln } \left[ {p\left[ {\frac{{\beta_{1} }}{{\alpha_{1} }}\left( {\frac{{t_{i} }}{{\alpha_{1} }}} \right)^{{\beta_{1} - 1}} \exp \left\{ { - \left( {\frac{{t_{i} }}{{\alpha_{1} }}} \right)^{{\beta_{1} }} } \right\}} \right]\left( {1 - p} \right)\left[ {\frac{{\beta_{2} }}{{\alpha_{2} }}\left( {\frac{{t_{i} }}{{\alpha_{2} }}} \right)^{{\beta_{2} - 1}} \exp \left\{ { - \left( {\frac{{t_{i} }}{{\alpha_{2} }}} \right)^{{\beta_{2} }} } \right\}} \right]} \right] \\ \;\;\;\; & \quad + (1 - \delta_{i} )\ln \left[ {\exp \left\{ { - \left( {\frac{{t_{i} }}{{\alpha_{2} }}} \right)^{{\beta_{2} }} } \right\} - p\left[ {\exp \left\{ { - \left( {\frac{{t_{i} }}{{\alpha_{2} }}} \right)^{{\beta_{2} }} } \right\} - \exp \left\{ { - \left( {\frac{{t_{i} }}{{\alpha_{1} }}} \right)^{{\beta_{1} }} } \right\}} \right]} \right] \\ \end{aligned}$$

The maximum likelihood estimates of the parameters are obtained by taking the partial derivatives of (13) with respect to *β*_1_, *α*_1_, *β*_2_, *α*_2_ and *p* and setting to zero. The maximum likelihood estimating equations obtained from (13) do not give closed form solutions for the parameters *θ* = {*β*_1_, *α*_1_, *β*_2_, *α*_2_, *p*}. Therefore, it requires a numerical iterative procedure for finding the MLEs of the parameters.

### Estimation of mixing proportions using EM Algorithm

The Expectation–Maximization (EM) algorithm is an efficient iterative procedure to compute the Maximum Likelihood Estimates (MLEs) of the parameters of the distribution in the presence of missing or hidden data (Dempster et al. 1977; McLachlan and Krishnan 2008). Bordes and Chauveau (2012) discussed several iterative methods based on EM and stochastic EM methodology to estimate parametric or semi parametric mixture models for randomly right censored lifetime data, conditioned that they are identifiable. Here we discuss the EM algorithm for finding the MLEs of the parameters of a general *K*-fold mixture model with parameters $\Theta = (p_{ 1} , \cdots , p_{K} , \theta_{ 1} , \cdots , \theta_{K} )$, where *p*_*j*_ is mixing parameters and *θ*_*j*_ is the parameters for the density function *f*_*j*_, $j = 1, 2, \ldots , K$. Let $y = \left( {t_{ 1} , \ldots , t_{n} } \right)^{\prime }$ denotes the observed random sample obtained from the mixture density. Let us introduce the unobservable or missing data vectors $z = \left( {z_{ 1}^{\prime } , \ldots , z_{n}^{\prime } } \right)^{\prime }$ , where *z*_*i*_ is a *K*-dimensional vector of zero–one indicator variables and where *z*_*ij*_ is one or zero according to whether*t*_*i*_arose or did not arise from the *j*-th component of the mixture $(i = 1, 2, \ldots , n; \;j = 1, 2, \ldots , K)$. The EM algorithm handles the unobservable data to the problem by working with the current conditional expectation of the complete-data log likelihood given the observed data. Let us define the complete-data vector *x* as $x = \left( {y^{\prime } , z^{\prime } } \right)^{\prime } .$

Each iteration of the EM algorithm consists of two steps. The Expectation or E-step computes the conditional expectation of the complete-data log-likelihood for *Θ* given observed data, which at the (*m* + 1)th iteration can be expressed as

14 $$Q\left( {\Theta ,\Theta^{\left( m \right)} } \right) = \sum\limits_{i = 1}^{n} {\sum\limits_{j = 1}^{K} {E_{{\Theta^{\left( m \right)} }} \left( {Z_{ij} \left| y \right.} \right)\partial_{i} \ln \left[ {p_{j}^{(m)} f_{j} \left( {t_{i} \left| {\theta_{j}^{(m)} } \right.} \right)} \right]} } + \sum\limits_{i = 1}^{n} {\sum\limits_{j = 1}^{K} {E_{{\Theta^{\left( m \right)} }} \left( {Z_{ij} \left| y \right.} \right)\left( {1 - \partial_{i} } \right)\ln \left[ {p_{j}^{(m)} R_{j} \left( {t_{i} \left| {\theta_{j}^{(m)} } \right.} \right)} \right]} }$$

As (14) is linear in the unobservable data *z*_*ij*_, the E-step (on the (*m* + 1)th iteration) simply requires the calculation of the current conditional expectation of *Z*_*ij*_ given the observed data *y*, where *Z*_*ij*_ is the random variable corresponding to *z*_*ij*_. Now

$$E_{{\Theta^{\left( m \right)} }} \left( {Z_{ij} \left| y \right.} \right) = z_{ij}^{\left( m \right)}$$

Here *z*_*ij*_^(*m*)^ are the posterior probabilities which can be expressed using the Bayes’s theorem as

15 $$z_{ij}^{\left( m \right)} = \frac{{p_{j}^{\left( m \right)} \left[ {\partial_{i} f_{j} \left( {t_{i} \left| {\theta_{j}^{\left( m \right)} } \right.} \right) + \left( {1 - \partial_{i} } \right)R_{j} \left( {t_{i} \left| {\theta_{j}^{\left( m \right)} } \right.} \right)} \right]}}{{\sum\limits_{j = 1}^{m} {p_{j}^{\left( m \right)} } \left[ {\partial_{i} f_{j} \left( {t_{i} \left| {\theta_{j}^{\left( m \right)} } \right.} \right) + \left( {1 - \partial_{i} } \right)R_{j} \left( {t_{i} \left| {\theta_{j}^{\left( m \right)} } \right.} \right)} \right]}}$$

The evaluation of this expectation is called the E-step of the algorithm. The second step (the Maximization or M-step) maximizes (14) with respect to the parameters to obtain new parameter estimations Θ^(*m*+1)^. To maximize (14), we can maximize the term containing *p*_*j*_ and the term containing *θ*_*j*_ independently since they are not related. To find the expression for *p*_*j*_, we introduce the Lagrange multiplier *λ* with the constraint $\sum\nolimits_{j = 1}^{K} {p_{j} = 1}$. Under this constraint, taking the derivative of (14) with respect to *p*_*j*_ and setting equal to zero, we get

16 $$\sum\limits_{i = 1}^{n} {\frac{1}{{p_{j} }}z_{ij}^{\left( m \right)} + \lambda = 0}$$

Summing both sizes over *j* and using $\sum\limits_{j = 1}^{m} {z_{ij}^{(m)} = 1}$; we get that *λ* = −*n* resulting in

17 $$p_{j}^{{\left( {m + 1} \right)}} = \frac{1}{n}\sum\limits_{i = 1}^{n} {z_{ij}^{\left( m \right)} }$$

See, Bucar et al. (2004) for more details.

For some distributions, it is possible to get closed-form analytical expressions for *θ*_*j*_. However, in the case of Weibull distributions with *θ*_*j*_ = (*α*_*j*_, *β*_*j*_), $j = 1,{ 2}, \ldots ,K$, we have to apply numerical procedures to find MLEs of the parameters. Here we apply the *survreg* function with weight (weight > 0) given in the *survival* package of the R-program. The algorithm proceeds by using the newly derived parameters as the guess for the next iteration. The E- and M-steps are iterated until the algorithm converges.

Finally, the EM algorithm for estimating the parameters of a *K*-fold Weibull mixture distribution can be summarized in a step-by-step procedure as follows:

- *Step 1* Begin with initial guesses of *p*_*j*_^(0)^, *α*_*j*_^(0)^ and *β*_*j*_^(0)^ for $j = 1, 2, \ldots , K$
- *Step 2* Using the initial values of *p*_*j*_^(0)^, *α*_*j*_^(0)^ and *β*_*j*_^(0)^, at *m*-th iteration calculate the conditional expectation of *z*_*ij*_, i.e., *z*_*ij*_^(*m*)^ using (15).
- *Step 3* At the*m*-th iteration, find the MLEs of *p*_*j*_^(*m*+1)^, *α*_*j*_^(*m*+1^ and *β*_*j*_^(*m*+1)^ as follows:
  - Find the MLE for *p*_*j*_^(*m*+1)^, using (17).
  - Estimate *α*_*j*_^(*m*+1)^, and *β*_*j*_^(*m*+1)^ using *survreg* function.
- *Step 4* Repeat Steps 2 and 3 until the algorithm converges with a desired accuracy.

The applications of the EM algorithm are broad because of its flexibility in analyzing incomplete or missing data. In any fields, when it is difficult to maximize the complicated likelihood function, various extensions and modifications of the EM algorithm have been proposed to simplify the computations, e.g., see Wei and Tanner (1990), Meng and Rubin (1993) and Liu and Rubin (1994). More detailed theory and applications of the EM algorithm can be found in McLachlan and Krishnan (2008).

## Case study: analysis of aircraft Windshield failure data

In this section, as a case study, we analyze a set of aircraft Windshield failure data. We apply twofold Weibull mixture model for the failure data and estimate various characteristics of the Windshield, such as the reliability function, B10 life, mean time to failure, etc. to assess the reliability of the Windshield.

### Aircraft Windshield failure data

Data on failure and censored times for a particular model of Windshield given in Table 1 are taken from Murthy et al. (2004), originally given in Blischke and Murthy (2000). The data consist of 88 failure times and 65 censored times out of 153 observations. Here censored time (or service time) means that the Windshields have not failed at the time of observation. The unit for measurement of time is 1000 h.

**Table 1:** Windshield failure data

*T*	*δ*	*T*	*δ*	*T*	*δ*	*T*	*δ*	*T*	*δ*
0.040	1	2.154	1	3.595	1	1.183	0	3.003	0
0.301	1	2.190	1	3.699	1	1.244	0	3.102	0
0.309	1	2.194	1	3.779	1	1.249	0	3.304	0
0.557	1	2.223	1	3.924	1	1.262	0	3.483	0
0.943	1	2.224	1	4.035	1	1.360	0	3.500	0
1.070	1	2.229	1	4.121	1	1.436	0	3.622	0
1.124	1	2.300	1	4.167	1	1.492	0	3.665	0
1.248	1	2.324	1	4.240	1	1.580	0	3.695	0
1.281	1	2.349	1	4.255	1	1.719	0	4.015	0
1.281	1	2.385	1	4.278	1	1.794	0	4.628	0
1.303	1	2.481	1	4.305	1	1.915	0	4.806	0
1.432	1	2.610	1	4.376	1	1.920	0	4.881	0
1.480	1	2.625	1	4.449	1	1.963	0	5.140	0
1.505	1	2.632	1	4.485	1	1.978	0		
1.506	1	2.646	1	4.570	1	2.053	0		
1.568	1	2.661	1	4.602	1	2.065	0		
1.615	1	2.688	1	4.663	1	2.117	0		
1.619	1	2.823	1	4.694	1	2.137	0		
1.652	1	2.890	1	0.046	0	2.141	0		
1.652	1	2.902	1	0.140	0	2.163	0		
1.757	1	2.934	1	0.150	0	2.183	0		
1.795	1	2.962	1	0.248	0	2.240	0		
1.866	1	2.964	1	0.280	0	2.341	0		
1.876	1	3.000	1	0.313	0	2.435	0		
1.899	1	3.103	1	0.389	0	2.464	0		
1.911	1	3.114	1	0.487	0	2.543	0		
1.912	1	3.117	1	0.622	0	2.560	0		
1.914	1	3.166	1	0.900	0	2.592	0		
1.981	1	3.344	1	0.952	0	2.600	0		
2.010	1	3.376	1	0.996	0	2.670	0		
2.038	1	3.385	1	1.003	0	2.717	0		
2.085	1	3.443	1	1.010	0	2.819	0		
2.089	1	3.467	1	1.085	0	2.820	0		
2.097	1	3.478	1	1.092	0	2.878	0		
2.135	1	3.578	1	1.152	0	2.950	0		

*T* times in 1000 h; δ failure/censored indicator

### Nonparametric estimate of reliability function

**Fig. 1:**
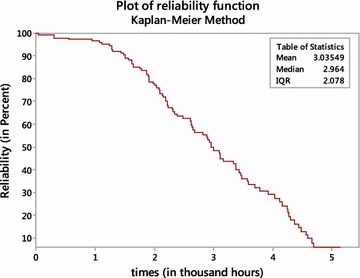
Nonparametric reliability plots

Figure 1 is the reliability (or survival) plot for the component. The plot appears to be reasonable; it shows the estimated MTTF is 3.03549 thousand hours or approximately 127 days. The nonparametric estimate of median lifetime is 2964 h, indicates that 50 % of the Windshield fails at 2964 h. The nonparametric estimate of cdf, known as empirical distribution function (edf) is one minus the estimated reliability function.

### Parametric estimate of reliability function

Murthy et al. (2004) assumed the twofold Weibull mixture model for this data set and estimated the model parameters based on Weibull Probability Plots (WPP) method. In this article we apply the EM algorithm, discussed in “Estimation of mixing proportions using EM algorithm”, to find the maximum likelihood estimates of the parameters *θ* = {*β*_1_, *α*_1_, *β*_2_, *α*_2_, *p*} for the twofold Weibull mixture model and investigate the performance of the proposed method over the method of Murthy et al. (2004). A comparison between the estimates of the parameters obtained by two different methods is given in Table 2.

**Table 2:** Estimates of parameters of twofold Weibull mixture model

Parameters	Estimates based on WPP	Estimates based on EM algorithm
$\hat{\beta }_{1}$	0.429	1.2098
$\hat{\alpha }_{1}$	8.230	0.2541
$\hat{\beta }_{2}$	2.990	2.7802
$\hat{\alpha }_{2}$	3.210	3.4856
$\hat{p}$	0.136	0.0176
$\left( {1 - \hat{p}} \right)$	0.864	0.9823

**Fig. 2:**
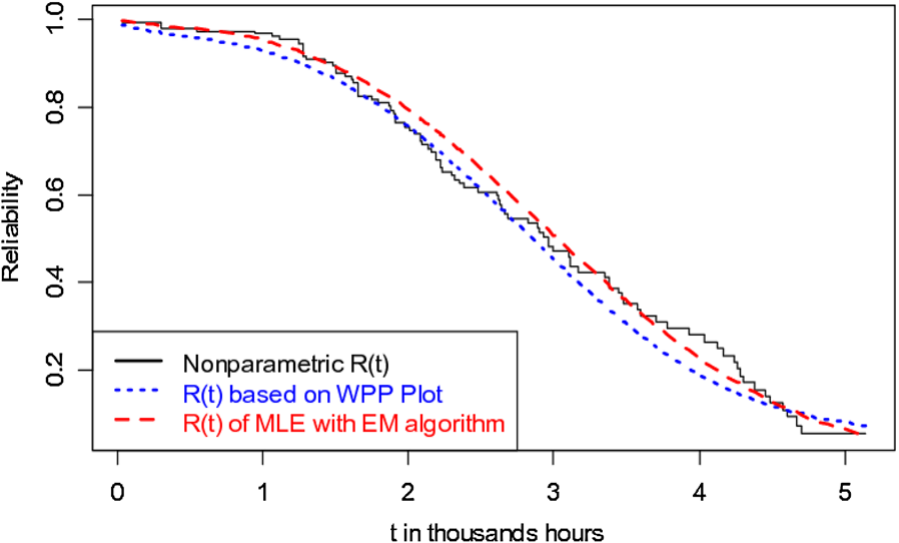
Comparison of reliability functions

**Fig. 3:**
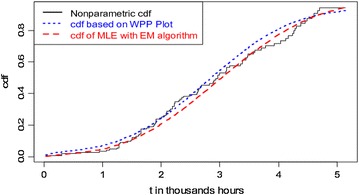
Comparison of cdfs

We have estimated the cdfs and reliability functions of twofold Weibull mixture model based on both non-parametric (by *Kaplan*–*Meier* method) and parametric (by EM Algorithm) approaches. The cdf and reliability function are also estimated by using the WPP plot method [estimates are taken from Murthy et al. (2004)]. Figures 2 and 3 compare the estimated reliability functions and cdfs, respectively to find out the best approach for the data set.

From Fig. 2, we observe that the reliability function obtained by the EM algorithm method is much closer to the Kaplan–Meier estimate than that of the reliability function estimated by the WPP plot method. The plots of cdfs shown in Fig. 3 conclude the same. These indicate that the method of estimation with the EM algorithm procedure is better than the WPP plot procedure.

The estimates of adjusted Anderson–Darling (AD) test statistic based on WPP method and EM algorithm method are 412.5845 and 410.2851, respectively. This again indicates that the EM algorithm method provides better fit for the data set than the WPP method.

### Reliability Characteristics of Windshield Data

Some of the reliability related important characteristics such as mean time to failure (MTTF), B10 lifetime, B50 (median) lifetime of the Windshield obtained by two methods are displayed in Table 3.

**Table 3:** Estimates of reliability characteristics of Windshield

Quantities	EM algorithm method	WPP Plot method
MTTF	3.0525	5.5782
B10-Lifetime	1.5248	1.3125
B50-Lifetime	3.0046	2.9298

Table 3 indicates that the estimates of MTTF obtained from maximum likelihood method via the EM algorithm and from WPP plot method are 3.0525 (thousand hours) and 5.5782 (thousand hours), respectively. Estimate of MTTF obtained by EM algorithm is very close to the nonparametric estimate of MTTF (3.03549 thousand hours) given in Fig. 1. The WPP method overestimates the MTTF in this case. From the estimates of B10-lifetime and B50-lifetime, we may conclude according to EM algorithm method that, 10 % of the total components fail approximately at 1524 h and 50 % fail at 3004 h.

## Simulation study

In this section, we use computer simulation to evaluate the performance of the method numerically. Numerically generated twofold mixture data are used to develop the twofold Weibull mixture model and to find the ML estimates of model parameters under right censored data. Using simulated data, the ML estimates of the model parameters, the sample means (SMs) and the mean squared errors (MSEs) of estimates are computed. Simulation programming codes are written using statistical software package R.

### Steps of simulation study

Here we describe the step-by-step algorithm for simulation of twofold Weibull mixture model and estimation of model parameters via the EM algorithm.

- *Step 1* We consider a set of true value for the 5 parameters *θ* = {*β*_1_, *α*_1_, *β*_2_, *α*_2_, *p*} of twofold Weibull mixture model. Under this set of parameter, we generate *n* = *n*_1_ + *n*_2_ samples from the twofold Weibull mixture model using the software R-Language (version-3.2.2). A desired percent (10, 20 and 30 %) of the largest generated sample out of 200, are considered as the right censored observations and remaining are assumed as failed lifetime.
- *Step 2* Based on the generated right censored data, we estimate the parameters via the EM algorithm assuming that the mixing sub-populations are unknown. The methodology is discussed in “Estimation of mixing proportions using EM algorithm”.
- *Step 3* The above Steps 1 and 2 are repeated 1000 times under two Cases:
- *Case (i)* for a variety percent of censored observations (10, 20 and 30 %) and
- *Case (ii)* for different sample sizes (*n* = 200, 400 and 600).
  We compute the sample means (SMs) and mean squared errors (MSEs) of the estimates for the both Cases (i) and (ii).
- *Steps4* Summarize and discuss the simulation results based on 1000 repetition.

### Simulation output analysis

The simulation results are shown in Tables 4 and 5.

**Table 4:** Sample means of the MLEs for different percent of censored observations

Parameters	True values	Sample means of the MLEs		
		Set-01 [N = 200; 10 % cens. obs.]	Set-02 [N = 200; 20 % cens. Obs.]	Set-03 [N = 200; 30 % cens. Obs.]
$\hat{\beta }_{1}$	3.50	3.7945	3.9587	4.1968
$\hat{\alpha }_{1}$	700.00	699.3536	700.1768	705.3863
$\hat{\beta }_{2}$	1.20	1.1601	1.1454	1.1709
$\hat{\alpha }_{2}$	850.00	944.5603	1055.9218	912.0363
$\hat{p}$	0.30	0.3737	0.3692	0.3732
$\left( {1 - \hat{p}} \right)$	0.70	0.6263	0.6308	0.6268

**Table 5:** Sample means of the MLEs for different sample sizes

Parameters	True values	Sample means of the MLEs		
		Set-04 [N = 200; 20 % cens. obs.]	Set-05 [N = 400; 20 % cens. obs.]	Set-06 [N = 600; 20 % cens. obs.]
$\hat{\beta }_{1}$	3.50	3.9587	3.8619	3.8365
$\hat{\alpha }_{1}$	700.00	700.1768	703.0983	702.2486
$\hat{\beta }_{2}$	1.20	1.1454	1.1664	1.1850
$\hat{\alpha }_{2}$	850.00	1055.9218	891.5291	873.1364
$\hat{p}$	0.30	0.3692	0.3398	0.3192
$\left( {1 - \hat{p}} \right)$	0.70	0.6308	0.6602	0.6808

Tables 4 and 5 present the summary results of the simulations based on 1000 repetitions under the given true values. In these tables, the first column shows the parameters of the model and second column shows the true values of the parameters. Tables 4 and 5 give the sample means of the MLEs of parameters obtained by the EM algorithm. For all of the sets, the sample means of the estimated parameters are close to the corresponding true values of the parameters. If the percent of censored observations decrease (i.e., if number of failures increase), the sample means of the MLEs become more closers to the true values for all most all sets, as expected. Similarly, the sample means of the MLEs become more closers to the true values for increasing sample sizes.

The mean squared errors (MSEs) of the MLEs of parameters for different percent of censored observations and for different sample sizes are given in Tables 6 and 7, respectively. The MSEs decrease for decreasing of the percent of censored observations (i.e., for increasing the number of failures). Also the MSEs decrease for increasing of the sample sizes. These comparisons indicate that the proposed method of estimation is applicable for analyzing mixture model for censored data.

**Table 6:** Mean squared errors for different percent of censored observations

Parameters	True values	Mean squared errors (MSEs) of MLEs		
		Set-01 [N = 200; 10 % cens. obs.]	Set-02 [N = 200; 20 % cens. obs.]	Set-03 [N = 200; 30 % cens. obs.]
$\hat{\beta }_{1}$	3.50	2.17953	2.6223	4.2156
$\hat{\alpha }_{1}$	700.00	3232.01855	4873.0835	9777.0312
$\hat{\beta }_{2}$	1.20	0.15011	0.0619	0.0711
$\hat{\alpha }_{2}$	850.00	87452.76431	1614229.0410	269,062.7034
$\hat{p}$	0.30	0.00114	0.0391	0.0500
$\left( {1 - \hat{p}} \right)$	0.70	0.03382	0.0391	0.0500

**Table 7:** Mean squared errors for different sample sizes

Parameters	True values	Mean squared errors (MSEs) of MLEs		
		Set-04 [N = 200; 20 % cens. obs.]	Set-05 [N = 400; 20 % cens. obs.]	Set-06 [N = 600; 20 % cens. obs.]
$\hat{\beta }_{1}$	3.50	2.6223	1.9544	1.5033
$\hat{\alpha }_{1}$	700.00	4873.0835	1907.8766	1574.6524
$\hat{\beta }_{2}$	1.20	0.0619	0.0294	0.0210
$\hat{\alpha }_{2}$	850.00	1614229.0410	25655.13287	10622.3852
$\hat{p}$	0.30	0.0391	0.0242	0.0159
$\left( {1 - \hat{p}} \right)$	0.70	0.0391	0.0242	0.0159

## Conclusion

There are situations where variations in product reliability can be occurred across different component vendors. In such situations, mixture of distributions can model the variability resulting from parts being bought from *K* different suppliers with *F*_*k*_(*t*) denoting the failure distribution for parts obtained from supplier *k*, $k = 1,{ 2}, \ldots ,K$. This paper has applied a twofold Weibull mixture model for analyzing product reliability data with failure and censored observations. It has proposed the Expectation–Maximization (EM) algorithm to find the maximum likelihood estimates of the parameters of mixture model and compared this method with a method based on Weibull Probability Paper plots. An aircraft component (Windshield) failure data is analyzed as an example and investigated that the performance of the proposed method of estimation is impressive. The results would be useful for managerial implications in assessing and predicting the reliability of the component more accurately.

The mixture model considered here is the twofold Weibull mixture model. The proposed method is easily extendable for other mixture models also. A scope of the future research with other mixture models and with various types of censored data would be interesting.

## Appendix

R codes for estimating parameters of Weibull–Weibull mixture model via the EM algorithm
